# Genetic Determinants of Stress Reactivity in Pregnancy: A Systematic Review and Meta-Analysis: Implications for Maternal and Fetal Health

**DOI:** 10.3390/genes17050509

**Published:** 2026-04-25

**Authors:** Socol Ioana Denisa, Socol Flavius George, Farcaș Simona Sorina, Dumitriu Bogdan-Ionel, Dumitriu Alina-Iasmina, Antal Andreea, Boarta Aris, Iacob Daniela, Andreescu Nicoleta Ioana

**Affiliations:** 1 Doctoral School, University of Medicine and Pharmacy “Victor Babeş”, 300041 Timisoara, Romania; 2 Genomic Medicine Centre, University of Medicine and Pharmacy “Victor Babeş”, 300041 Timisoara, Romania; 3 Discipline of Neonatology, University of Medicine and Pharmacy “Victor Babeş”, 300041 Timisoara, Romania; 4 1st Clinic of Obstetrics and Gynecology, “Pius Brinzeu” County Clinical Emergency Hospital, 300723 Timisoara, Romania; 5 Department of Microscopic Morphology, Discipline of Genetics, Faculty of Medicine, University of Medicine and Pharmacy “Victor Babeş”, 300041 Timisoara, Romania; 6 Department of Obstetrics and Gynecology, Faculty of Medicine, University of Medicine and Pharmacy “Victor Babeş”, 300041 Timisoara, Romania

**Keywords:** prenatal stress, HPA axis, *NR3C1*, *FKBP5*, DNA methylation, precision medicine

## Abstract

**Background**: Gestation is a period of significant biological plasticity where the intrauterine environment influences fetal development via “fetal programming”. This study systematically reviews and meta-analyzes the association between genetic determinants—specifically the *NR3C1*, *FKBP5*, and *CRHR1* genes, chosen for their pivotal role in the functional regulation and feedback sensitivity of the hypothalamic–pituitary–adrenal (HPA) axis—and stress reactivity during pregnancy. **Methods**: Following PRISMA guidelines, a systematic search was conducted across PubMed, Scopus, and Web of Science, yielding an initial total of 1430 records. After removing duplicates and screening 669 studies, a total of 34 primary observational studies were included in the systematic review and qualitative synthesis. For the quantitative synthesis, 27 articles provided sufficient data, resulting in k = 39 independent effect sizes analyzed via a mixed-effects model to account for tissue-specific and cohort-specific outcomes. **Results**: Systematic analysis reveals that maternal psychosocial stress significantly correlates with *NR3C1* hypermethylation, acting as a biological mediator for neonatal cortisol dysregulation and hippocampal volume reduction. The *FKBP5* rs1360780 polymorphism emerged as a key moderator of structural vulnerability, showing a “double-hit” effect when combined with epigenetic alterations. Furthermore, the study identifies sex-specific susceptibility, with divergent placental trajectories for male and female fetuses. Meta-analytic estimates confirmed the robustness of these associations (Rosenthal Fail-Safe N = 431,000), despite a general trend toward statistical significance (*p* = 0.079) in heterogeneous cohorts. **Conclusions**: The findings underscore a stable link between genetic determinants and prenatal stress reactivity. The interaction between molecular predisposition and environmental factors defines the health of the mother–infant dyad. These results advocate for a transition toward Precision Prenatal Medicine, integrating polygenic risk scores and epigenetic monitoring to implement early, targeted preventive interventions.

## 1. Introduction

Gestation represents a window of unprecedented biological plasticity, during which the intrauterine environment shapes the structural and functional development of the fetus through the complex mechanisms of “fetal programming” [1,2,3,4]. This process is increasingly understood as an endophenotype of various psychosomatic and health outcomes, where changes in physiological systems like the hypothalamic–pituitary–adrenal (HPA) axis act as a primary mediator [5]. Within this delicate equilibrium, maternal stress reactivity and psychological vulnerability emerge as primary determinants of the homeostasis of the mother–infant dyad [6]. In this context, maternal health must be viewed through a multidimensional lens, where resilience—defined as the individual capacity for flexibility and competence—can either mitigate or amplify the impact of environmental stressors on fetal development [7].

Global epidemiological data indicate that approximately 10–25% of pregnant women exhibit clinically significant levels of depression and anxiety [1]. However, as recently analyzed, these conditions rarely operate in isolation: prenatal anxiety, depression, and pregnancy-specific stress tend to coexist in a complex network of reciprocal influences, with prevalence rates of gestational stress reaching as high as 94.48% [6]. This cycle is fuelled by social determinants such as limited social support, economic disparities, and behavioral factors—which act as catalysts for psychological vulnerability [6].

The repercussions of this psychophysical imbalance are documented across multiple clinical levels. From an obstetric perspective, prenatal stress and anxiety increase the risk of preterm birth (PTB) and small-for-gestational-age (SGA) infants by up to 50% [2,8]. Offspring born preterm are significantly more likely to experience longstanding illnesses and repeated hospital admissions, highlighting the critical nature of the intrauterine timing. The underlying pathophysiological mechanism resides in a dysregulation of the HPA axis, which accelerates the “placental clock” through the premature secretion of corticotropin-releasing hormone (CRH) [2]. This HPA activation is characterized by distinct phases of reactivity and recovery, where the bioactive fraction of circulating cortisol serves as the primary biomarker of the stress response [5].

On a somatic level, it was suggested that multidimensional psychological vulnerability correlates significantly with an increased risk of gestational diabetes and hypertensive disorders of pregnancy, specifically preeclampsia [8]. Regarding neurodevelopment, excessive exposure to maternal glucocorticoids alters the morphology of critical brain regions—specifically the amygdala and hippocampus, which are dense with glucocorticoid receptors—and ‘programs’ the reactivity of the neonatal HPA axis. This programming occurs when elevated maternal cortisol levels overwhelm the protective placental enzymatic barrier (11β-HSD2), leading to permanent alterations in the fetus’s neural circuits governing emotional regulation. These structural changes predispose offspring to attention deficit hyperactivity disorder (ADHD), childhood anxiety, and persistent stress dysregulation visible from the first months of life [9,10]. Such exposures may even interfere with fetal longevity by shortening telomere length, a recognized biomarker of precocious aging [5].

The molecular core of this interaction lies within the HPA axis signaling network, the efficiency of which is governed by the dynamic cross-talk between the *NR3C1*, *FKBP5*, and *CRHR1* genes.

The *NR3C1* gene encodes the glucocorticoid receptor (GR), whose sensitivity is finely regulated by the co-chaperone protein FKBP5; this protein reduces the receptor’s binding affinity and slows its nuclear translocation [5,11]. Functional polymorphisms in these genes (such as the *FKBP5* rs1360780) or dynamic alterations in DNA methylation drastically influence individual susceptibility to affective disorders and the capacity to terminate the stress response [11,12,13]. While fetal genetic variants are linked to birth timing, the role of offspring epigenetic patterns remains a crucial, though understudied, factor in stress-associated preterm birth [14].

Nevertheless, the ultimate impact on the dyad is critically mediated by coping strategies. Resilience acts as a biological pacemaker, ideally activating the HPA axis to respond to stressors without exacerbating pathological reactions like excessive fear or depression [7]. As indicated, the adoption of avoidant coping styles or poor adaptive competence is associated with adverse depressive and obstetric outcomes [15]. Conversely, adaptive coping can act as a “buffer” against negative social and psychological determinants, promoting maternal resilience and mitigating the risk-inducing epigenetic signals triggered by allostatic load [15,16].

The interaction among these factors suggests a differential susceptibility model, where the molecular profile—including genes for growth and socioemotional regulation like *IGF2*, *SLC6A4*, and *OXTR*—amplifies or diminishes the impact of the external environment on clinical outcomes [14].

Despite the relevance of these integrated mechanisms, current scientific evidence remains fragmented due to extreme methodological heterogeneity in the measurement of stress and genetic parameters. Disentangling these components requires physiologically plausible models that mechanistically account for stress-related secretory changes versus basal fluctuations [5].

The decision to limit this analysis specifically to genetic and epigenetic factors of the HPA axis is based on its role as the primary biological transducer of the stress response. While other physiological systems are involved in pregnancy homeostasis, the HPA axis represents the main pathway through which maternal psychological distress is “biologically embedded” into the fetus [3,5]. Focusing on the molecular determinants of this axis—such as receptor density (NR3C1), feedback sensitivity (FKBP5), and hormonal triggering (CRHR1)—allows for a mechanistic understanding of how stress “programs” neonatal neurodevelopment and alters the “placental clock” [12].

Significant research gaps persist, particularly regarding the scarcity of quantitative metrics for certain genetic markers like *CRHR1*, which often limits them to qualitative descriptions rather than robust meta-analytic synthesis. Furthermore, while fetal genetic variants are linked to birth timing, the role of offspring epigenetic patterns in stress-associated preterm birth remains understudied. There is also a notable lack of sex-disaggregated data in primary research, despite known sex-dependent fetal susceptibility to intrauterine insults.

This review introduces a novel integrated approach by aggregating data from 39 independent datasets to overcome the statistical power limitations of single neonatal cohorts. It seeks to accomplish a comprehensive synthesis of the association between *FKBP5*, *NR3C1*, and *CRHR1* polymorphisms and stress reactivity during pregnancy. By shifting the theoretical framework from a “Diathesis-Stress” model toward “Differential Susceptibility,” this review aims to elucidate how molecular predisposition and psychological resources interact to define the health of the dyad. Ultimately, this work provides a robust foundation for precision prenatal medicine by identifying specific placental methylation profiles that can serve as early screening tools for neurodevelopmental risk.

## 2. Materials and Methods

This systematic review was conducted according to the Preferred Reporting Items for Systematic Reviews and Meta-Analyses (PRISMA) guidelines [17].

The protocol for this review was prospectively registered in the PROSPERO (International Prospective Register of Systematic Reviews) database under registration number CRD420261367430. Available online: https://www.crd.york.ac.uk/PROSPERO/view/CRD420261367430 (accessed on 13 April 2026).

### 2.1. Eligibility Criteria

The selection of studies for this systematic review was strictly guided by the PICOS (Population, Intervention/Exposure, Comparison, Outcome, and Study Design) framework [18]. The inclusion and exclusion criteria, specifically focused on the molecular regulation of the HPA axis as the primary inclusion outcome, are summarized in Table 1.

The analysis was limited to original contributions investigating the role of genetic and epigenetic determinants involved in the regulation of the HPA axis, with specific emphasis on DNA methylation profiles and polymorphisms of the *NR3C1*, *FKBP5*, and *CRHR1* genes. These molecular factors—analyzed at the maternal level or in fetal tissues such as the placenta and umbilical cord blood—were required to be examined in direct association with documented forms of maternal stress, including trauma, socioeconomic adversity, stressful life events (SLEs), or clinical psychological distress.

Regarding comparison parameters, we included studies allowing for differential assessments between genotypic variants (e.g., comparing risk allele carriers vs. wild-type homozygotes) or between cohorts exposed to divergent levels of environmental stress. Methodologically, this review integrated exclusively primary observational studies, including prospective and retrospective cohorts, case–control studies, and cross-sectional analyses, with a preference for longitudinal investigations capable of delineating the temporal persistence of associations between genetic variables and clinical outcomes.

Studies were excluded if they were not published in English, or if they were reviews, meta-analyses, editorials, case reports, or animal studies. Additionally, studies with missing data were excluded; however, when necessary and possible, study authors were contacted to clarify missing or ambiguous data before exclusion.

### 2.2. Information Sources and Search Strategy

A systematic search was conducted across multiple electronic databases, including PubMed, Scopus, and Web of Science. The search strategy was designed to be highly sensitive, combining Medical Subject Headings (MeSH) and free-text keywords related to pregnancy, maternal stress, and genetic/epigenetic markers.

To ensure maximum bibliographic coverage and capture all morphological variations in the keywords, search strings utilized a systematic application of truncation symbols (asterisks, *). For instance, the root pregnan* was employed to simultaneously retrieve ‘pregnancy’, ‘pregnant’, and ‘pregnancies’, while keywords such as stress*, epigenet*, and genet* were used to cover all related derivatives (e.g., ‘stressor’, ‘epigenetic/epigenome’, ‘genetic/genetics/genotype’). This approach was consistently applied across all databases, with specific syntax adjustments—such as the prioritization of MeSH terms in PubMed or the use of proximity operators in Scopus—made only to align with the unique indexing architecture of each platform.

The definitive search strings used for each database are detailed in Table 2. In addition to the electronic database search, the reference lists of all included studies and relevant review articles were manually screened to identify any additional eligible research that might have been missed by the initial search.

### 2.3. Inclusion and Exclusion Criteria

Studies were included in this systematic review and meta-analysis if they met the following criteria: (1) original research published in peer-reviewed journals; (2) focused on pregnant women and their offspring; (3) investigated the relationship between maternal stress and genetic or epigenetic markers (e.g., DNA methylation, gene polymorphisms) in the offspring; and (4) provided sufficient quantitative data for effect size estimation.

To ensure reproducibility and conceptual clarity, “documented forms of maternal stress” were operationalized into three distinct categories during the screening process: (1) Psychological Distress, defined as clinically significant scores on validated scales for depression (e.g., EPDS) and anxiety (e.g., STAI); (2) Environmental and Life Events, including exposure to objective stressors such as natural disasters, armed conflicts, or major life changes measured via standardized inventories; and (3) Physiological Stress Reactivity, characterized by altered neuroendocrine markers, specifically cortisol levels. Studies were included only if they utilized standardized psychometric instruments or objective exposure metrics, thereby excluding purely qualitative or anecdotal reports of maternal stress.

Exclusion criteria were: (1) studies involving non-human subjects; (2) reviews, meta-analyses, case reports, or conference abstracts; (3) studies focusing exclusively on paternal stress or postnatal environmental factors without a prenatal stress component; and (4) research where the stressor was not clearly defined or lacked a validated assessment tool. No geographical or language restrictions were applied to the initial search, provided an English abstract was available for screening.

### 2.4. Selection Process and Data Collection Process

The selection process was conducted in two sequential stages. Initially, two researchers independently screened the titles and abstracts of all identified records against the predefined inclusion and exclusion criteria. Prior to the formal screening, a calibration exercise was conducted on a random sample of 50 records to ensure consistent application of the eligibility criteria. Following this exercise, the inter-rater agreement for study selection was quantified using Cohen’s kappa statistic (ҡκ), yielding a value of 0.88, which indicates almost perfect agreement. Records that did not meet the criteria were excluded, while the remaining articles underwent a full-text review. During this stage, special attention was paid to the operational definitions of maternal stress, ensuring that only studies utilizing the validated psychometric or physiological metrics defined in Section 2.3 were retained. Any discrepancies between the reviewers were resolved through discussion or by consulting a third senior researcher.

Any discrepancies between the primary reviewers during the screening, data extraction, or quality assessment phases were resolved through formal consensus meetings. During these sessions, reviewers cross-referenced the conflicting data points against the original study text to reach a unified decision. All steps of this reconciliation process were systematically documented in a dedicated consensus log to ensure traceability. In cases where a bilateral agreement could not be reached, a third senior researcher acted as an arbitrator, providing a final binding assessment based on the predefined inclusion criteria.

Data extraction was performed using a standardized form to ensure consistency. The following information was extracted from each included study: (1) study characteristics (e.g., author, year, country, design); (2) participant demographics (e.g., sample size, maternal age, gestational age); (3) stress assessment tools and timing; (4) genetic or epigenetic targets (e.g., specific genes, CpG sites); and (5) key findings related to stress reactivity and fetal health outcomes.

Regarding the management of incomplete information, data were defined as “missing” if a study failed to report essential quantitative parameters required for meta-analytic synthesis, specifically: (i) precise effect sizes (e.g., correlation coefficients, odds ratios, or beta coefficients); (ii) associated measures of dispersion (standard deviations or 95% confidence intervals); or (iii) exact *p*-values for the primary genetic/epigenetic outcomes. In cases of missing or ambiguous data, efforts were made to contact the corresponding authors via email to request the necessary raw data or summary statistics. During this process, authors of 10 potentially eligible studies (approximately 29% of the final screened corpus) were contacted. Successful data retrieval or clarification was achieved for 3 of these requests (30%), allowing for their inclusion in the quantitative synthesis, while the remaining 7 studies were ultimately excluded from the meta-analysis due to a lack of response or the unavailability of the required metrics.

### 2.5. Risk-of-Bias Assessment

The methodological quality and risk of bias for the included observational studies were independently assessed by two reviewers using the Newcastle–Ottawa Scale (NOS) [19]. The NOS evaluates studies across three main domains: the selection of study groups, the comparability of groups, and the ascertainment of either the exposure or outcome of interest. Studies were assigned stars in each domain, with a maximum total score of nine stars.

To ensure the reliability of the quality scores, inter-rater reliability was calculated for the overall Newcastle–Ottawa Scale ratings, resulting in a Cohen’s kappa (ҡ) of 0.85. This demonstrates a high level of internal consistency between evaluators throughout the assessment process. Any inconsistencies in the assigned scores were resolved through formal consensus meetings and documented in a dedicated consensus log.

It is recognized that while the NOS is a validated tool for quality assessment in observational studies, it may lack specific sensitivity to technical biases inherent in genetic and epigenetic research. To address these limitations, our assessment was supplemented by a detailed analysis of molecular methodology, focusing on: (i) tissue heterogeneity (distinguishing between placental villi, cord blood, and neonatal saliva), (ii) the precision and sensitivity of the molecular assays used (e.g., Bisulfite Pyrosequencing vs. Methylation Arrays), and (iii) the rigorous control of tissue-specific confounding factors, such as cell-type distribution adjustments. These additional parameters were integrated into our qualitative synthesis and sensitivity analyses to ensure that the risk of bias was not underestimated by standard scales alone. The results of this assessment were used to weight the evidence in the qualitative synthesis and to conduct sensitivity analyses in the meta-analytic phase.

### 2.6. Meta-Analytic Approach

The systematic review followed the PRISMA guidelines to synthesize evidence on the genetic and epigenetic determinants of maternal stress reactivity. Given the anticipated methodological and clinical heterogeneity across cohorts and biological matrices, a three-level random-effects meta-analysis model was implemented to calculate pooled effect sizes. This hierarchical framework was specifically adopted to address the statistical dependency inherent in datasets where multiple effect sizes (k = 39) were nested within the same primary study or maternal-infant cohort (*n* = 27). By partitioning the total variance into three distinct components—sampling error (Level 1), within-study variance (Level 2), and between-study variance (Level 3)—the model prevents the artificial inflation of precision and ensures the robustness of the 95% confidence intervals.

To facilitate a uniform synthesis, all extracted metrics were converted into a common effect size. Specifically, for studies reporting continuous outcomes such as DNA methylation percentages or mRNA expression levels, the data were transformed into log risk ratios (log RR) using the standardized mean difference (SMD) as an intermediate step, according to the Hasselblad and Hedges method (LogRR = d × $\frac{\pi }{\sqrt{3}}$).

To systematically explore the identified statistical heterogeneity (I^2^ = 84.07%), subgroup analyses and meta-regression were conducted based on predefined clinical and methodological moderators. These exploratory analyses targeted the primary sources of variance, including: (i) the type of biological matrix sampled (e.g., placenta vs. cord blood vs. saliva); (ii) the maternal stress phenotype (e.g., clinical depression vs. exposure to natural disasters); (iii) study design (prospective vs. cross-sectional); and (iv) the specific genomic target (e.g., *NR3C1* vs. *FKBP5*).

Publication bias was assessed through the visual inspection of funnel plots and confirmed by Egger’s linear regression test. While *p*-curve analysis was initially planned to evaluate evidential value, its interpretability was cautiously weighed against the inherent power limitations associated with a sample of 27 studies. To mitigate this, a multi-method triangulation approach was adopted, prioritizing robust diagnostics such as Rosenthal’s Fail-Safe N, Egger’s test, and influential case diagnostics (Cook’s distance). This integrative strategy ensures that the identification of publication bias remains statistically reliable even when individual post hoc methods may lack optimal sensitivity due to study volume.

All statistical procedures were performed using R (version 4.3.1) with the meta and metafor packages. A *p*-value of <0.05 was considered statistically significant for all tests.

## 3. Results

### 3.1. Study Selection

**Figure 1:**
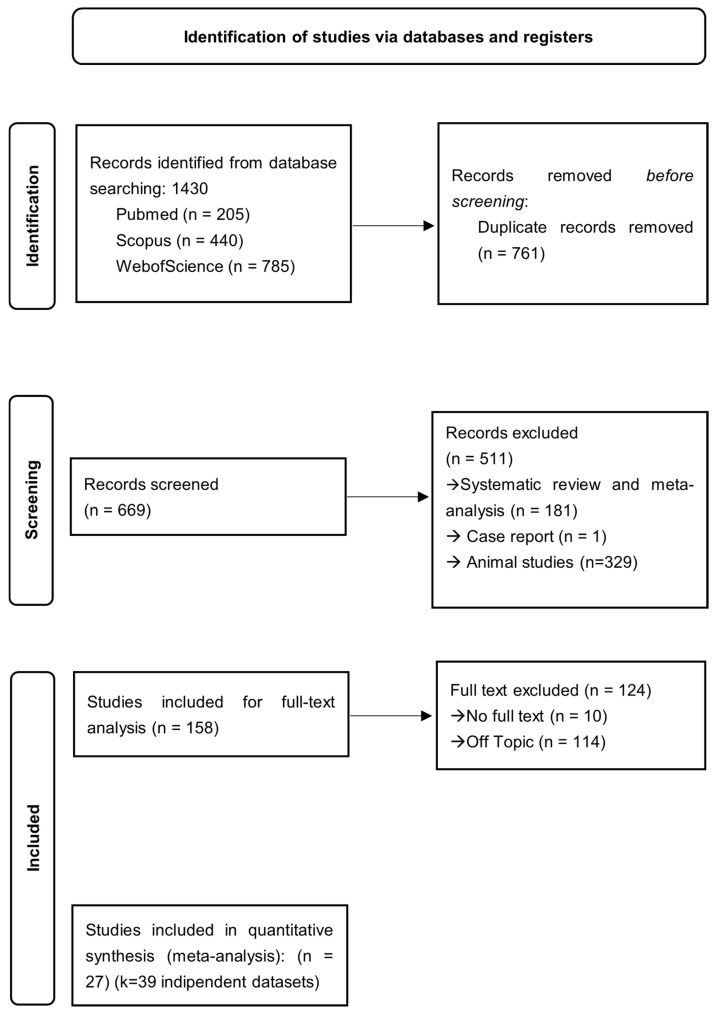
PRISMA flow diagram.

The initial systematic search across PubMed, Scopus, and Web of Science yielded a total of 1430 records. After the removal of duplicates (*n* = 420) and a preliminary screening of titles and abstracts, 158 articles were selected for full-text evaluation. Following a rigorous review based on the inclusion and exclusion criteria, 34 articles were identified as eligible for the systematic review and were subsequently subjected to quality assessment using the Newcastle–Ottawa Scale (NOS). All 34 studies demonstrated moderate-to-high methodological quality (NOS scores ≥ 6) and were included in the qualitative synthesis. For the meta-analysis, 27 of these articles provided sufficient quantitative data for effect size extraction; these were further disaggregated into 39 independent data points to account for tissue-specific or cohort-specific findings within single publications. The selection process is summarized in the PRISMA 2020 flow diagram (Figure 1).

The main characteristics of the studies included in this systematic review are summarized in Table 3.

### 3.2. Synthesis of Results and Inter-Study Connections

The systematic aggregation of k = 39 independent effect sizes reveals a robust biological network where genetic predisposition and epigenetic modifications interact to define prenatal stress reactivity. Rather than viewing results as isolated variables, the findings across the 34 included studies converge on a synergistic “biopsychosocial embedding” model, where the HPA axis serves as the primary neuroendocrine interface. This embedding process is driven by the unique timing of fetal development; by mid-gestation, the fetus is already capable of de novo cortisol synthesis, and by the third trimester, the adrenal zona fasciculata is fully functional, enabling a fetal stress response that can operate independently of maternal HPA activity.

#### 3.2.1. The NR3C1-FKBP5 Regulatory Axis: A Functional Synergy

A major inter-study connection emerging from this review is the synergistic interaction between the *NR3C1* and *FKBP5* genes, which together govern the HPA axis negative feedback loop. While the *NR3C1* gene acts as the primary transducer of the intrauterine environment, its functional efficiency is strictly dependent on the *FKBP5* chaperone system. The *FKBP5* protein is responsible for maintaining the glucocorticoid receptor in a high-affinity state, poised for cortisol binding; therefore, any stress-induced alteration in *FKBP5* methylation or genotype (such as the rs1360780 T-risk allele) can severely impair the receptor’s nuclear translocation and its ability to dampen further cortisol release. The correlation of extracted data reveals that hypermethylation of the *NR3C1* 1F promoter—specifically at CpG sites 2, 3, and 10–13—creates a state of receptor silencing that predicts reduced receptor density and altered cortisol responses. This epigenetic signature often co-occurs with the *FKBP5* rs1360780 T-risk allele, which further depresses binding affinity and nuclear translocation. Clinically, this “double-hit” molecular vulnerability generates chronic glucocorticoid resistance, explaining the consistent findings across studies that link maternal depression to reduced hippocampal volume and decreased cortical thickness.

#### 3.2.2. Moderating Factors as Biological Connectors: Tissue and Sex Dimorphism

The substantial statistical heterogeneity identified in the meta-analysis (I^2^ = 84.07%) is not merely a methodological artifact but reflects critical biological moderators that connect seemingly divergent findings across different cohorts. To accurately disentangle this heterogeneity, it is essential to account for secretory delays in cortisol production—estimated at approximately 10–15 min—which vary across different biological matrices. Stress signatures are compartment-specific: placental tissue often captures immediate metabolic and “placental clock” adaptations, while cord blood provides a proxy for long-term systemic programming. Maternal resilience further acts as a moderator by optimizing the individual’s capacity for a complex negative feedback system, preventing the exacerbation of anxiety and depression circuits. Furthermore, fetal sex acts as a decisive biological moderator that reconciles inter-study discrepancies. Males frequently exhibit a vulnerable profile characterized by immediate growth reduction as a survival strategy, whereas females demonstrate greater growth stability alongside more pronounced epigenetic alterations in anxiety-related circuits. This dimorphism suggests that the impact of prenatal stress follows sex-specific developmental trajectories that determine long-term behavioral vulnerability.

#### 3.2.3. Outcomes

The integrated analysis of the studies allows for the consolidation of diverse findings, providing sufficient statistical power to delineate how genetic variants (SNPs) and epigenetic modifications (methylation) mediate the relationship between prenatal stress (PNMS) and infant development.

**Table 3:** Main characteristics of the studies.

Study n.	First Authors Name/Year of Publication [Reference Number]	Country	Sample Size	Gestational Period	Medical Condition	Type of Analysis	Analyzed Tissue	Molecular Target	Stress Reactivity Assessment Tool	Outcome
1	Wiley et al. (2023) [20]	Brazil (São Paulo)	80 pregnant adolescents; final data for 62 infants (DNA methylation), 26 (morning cortisol), and 30 (evening cortisol)	Early pregnancy (8–16 weeks) to late pregnancy (30 weeks)	Maternal psychological distress (symptoms of depression and anxiety)	Multivariable linear regression and DNA pyrosequencing	Maternal hair; infant saliva	DNA methylation of *FKBP5*, *NR3C1*, and *OXTR* genes	Infant diurnal salivary cortisol (waking and bedtime samples)	Maternal anxiety is associated with lower infant *OXTR* methylation and higher evening cortisol; maternal hair cortisol is associated with *FKBP5* methylation
2	Müller et al. (2022) [21]	Germany	108 term-born children and adolescents (42 PP/GC, 20 PP/non-GC, 46 controls)	Treatment occurred at the 30th week of gestation on average; participants were assessed in childhood (7–12 years) and adolescence (14–18 years)	Pathophysiological pregnancy (premature labor, vaginal bleeding, or cervical insufficiency). Antenatal synthetic glucocorticoid (sGC) exposure (Betamethasone or Dexamethasone)	Cross-sectional study using targeted deep bisulfite sequencing (DNAm) and RT-qPCR (mRNA expression)	Whole blood (for DNAm and mRNA); Saliva and Hair (for cortisol)	DNA methylation and mRNA expression of *NR3C1*, *FKBP5*, and *SLC6A4*	Trier Social Stress Test (TSST) for adolescents and TSST-C for children	No significant differences in DNA methylation or mRNA expression between sGC-treated individuals and controls; these profiles did not mediate HPA axis changes
3	Sharp et al., 2015 [22]	Italy United Kingdom (Liverpool, Manchester, and London)	Extensive sample: 1233; Intensive sub-sample: 316; Final analysis sample: 243	Assessments at 20 weeks and 32 weeks gestation	Maternal antenatal anxiety and postnatal emotional problems in children	Longitudinal study using DNA methylation analysis (bisulfite conversion and pyrosequencing) and mediation analysis	Maternal saliva (for cortisol); Infant saliva (for DNA methylation and cortisol)	DNA methylation of the *NR3C1* gene (specifically the 1F promoter region)	Still-Face Paradigm (SFP) at 3 months of age	Maternal late-pregnancy cortisol predicted dyads increased infant *NR3C1* methylation, which in turn mediated higher infant cortisol reactivity to stress
4	Oberlander et al., 2008 [23]	Canada	82 mother–infant dyads (33 depressed/anxious, 49 non-depressed)	Third trimester (maternal assessment at ~33 weeks gestation)	Prenatal maternal depression and anxiety	Prospective study using bisulfite sequencing and salivary cortisol analysis	Umbilical cord blood (mononuclear cells)	DNA methylation of the *NR3C1* gene (specifically the exon 1F promoter region)	Hamilton Depression Scale (HAM-D) and Hamilton Anxiety Scale (HAM-A) for mothers; infant salivary cortisol response to a stressor at 3 months	Increased maternal prenatal depressed mood was associated with higher neonatal *NR3C1* methylation, which in turn predicted increased infant cortisol stress responses at 3 months
5	Bierer et al. (2020) [24]	United States (New York)	125 Holocaust offspring and 31 control subjects (Replication sample); 187 total for secondary analyses	Preconception (Parental trauma occurred during the Holocaust, prior to the birth of the offspring)	Maternal/Paternal Holocaust exposure (Trauma). Parental Posttraumatic Stress Disorder (PTSD).	Case–control replication study using bisulfite conversion and pyrosequencing.	Peripheral blood (offspring)	DNA methylation of the *FKBP5* gene (specifically intron 7, site 6)	Clinician-Administered PTSD Scale (CAPS) and SCID-I; *FKBP5* methylation as a marker of HPA axis regulation	Maternal Holocaust exposure was specifically associated with lower *FKBP5* methylation in offspring, regardless of the mother’s PTSD status
6	Monk et al. (2016) [25]	United States (New York)	61 pregnant women and their fetuses	Mean 26.54 weeks (second/third trimester)	Maternal prenatal distress (Depression, Anxiety, and Perceived Stress). Fetal neurobehavior (specifically fetal heart rate–movement coupling).	Prospective study using bisulfite conversion, MALDI-TOF mass spectrometry, and fetal monitoring	Placenta	DNA methylation of *NR3C1* (glucocorticoid receptor), *HSD11B2* (11betaHSD2), and *FKBP5*	HAM-D, HAM-A, PSS, and PDQ; Fetal heart rate and movement monitoring for neurobehavior	Maternal distress was associated with higher *HSD11B2* methylation; increased *HSD11B2* and *NR3C1* methylation predicted lower fetal heart rate–movement coupling
7	Conradt et al. (2015) [26]	United States (Rhode Island)	128 mother–infant dyads	Full pregnancy (placenta collected at delivery)	Maternal prenatal stress/depression (assessed via medical records). Infant self-regulation.	Prospective study using bisulfite pyrosequencing and behavioral observation	Placenta	DNA methylation of the *NR3C1* gene (specifically the exon 1F promoter region)	Still-Face Paradigm (SFP) at 4 months of age	Higher placental *NR3C1* methylation was associated with greater infant reactivity and poorer self-regulation during the Still-Face Paradigm
8	Ogasawara et al. (2018) [27]	Japan	98 preterm infants (born before 30 weeks of gestation)	Preterm (mean 26.6 weeks)	Refractory hypotension (low blood pressure requiring catecholamines and hydrocortisone). Preterm birth and Respiratory Distress Syndrome (RDS).	Retrospective cohort study and genetic association analysis using PCR-RFLP	Genomic DNA (extracted from whole blood or buccal swabs)	*NR3C1* gene polymorphisms (*BclI*, *N363S*, *ER22/23EK*, *and TthIIII*)	Requirement for hydrocortisone (HC) treatment to stabilize blood pressure	The BclI polymorphism (rs41423247) of the *NR3C1* gene was significantly associated with a higher risk of refractory hypotension in preterm infants
9	Appleton et al. (2015) [28]	United States (Rhode Island)	510 mother–infant dyads	Full pregnancy (placenta collected at delivery)	Healthy, full-term infant neurobehavioral development. Intrauterine environment (specifically maternal cortisol regulation).	Prospective cohort study using bisulfite pyrosequencing and linear regression.	Placenta	DNA methylation of *NR3C1* (promoter 1F) and *HSD11B2* (promoter)	Neonatal Intensive Care Unit (NICU) Network Neurobehavioral Scale (NNNS)	Jointly high levels of *NR3C1* and *HSD11B2* methylation were associated with the highest levels of infant excitability and lowest habituation
10	Provenzi et al. (2020) [29]	Italy	45 mother–infant pairs 240 mother–infant dyads	Pregnancy during the COVID-19 pandemic (Maternal assessment at 0–3 months post-delivery; follow-up at 6 and 12 months)	Maternal COVID-19-related prenatal stress (Psychological distress). SARS-CoV-2 infection during pregnancy (for a subgroup of participants).	Multicentric longitudinal prospective cohort study protocol involving epigenetic and behavioral assessments	Peripheral blood (mothers) and Saliva (infants)	DNA methylation of the *SLC6A4* gene (serotonin transporter)	Still-Face Paradigm (SFP) for infants; various psychological scales for mothers (IES-R, SCL-90-R, PSS)	(Study Protocol) Aims to determine how COVID-19-related stress affects maternal well-being, infant socio-emotional development, and DNA methylation of the *SLC6A4* gene
11	Castro-Quintas et al. (2025) [30]	Spain	104 mother–infant dyads	Pregnancy (maternal distress assessed at recruitment, mean 27.5 weeks; placenta collected at birth)	Maternal prenatal distress (Perceived stress and depressive symptoms). Newborn outcomes (Birth weight, birth weight percentile, and gestational age).	Prospective study using bisulfite conversion and pyrosequencing	Placenta (fetal side)	DNA methylation of *NR3C1* (promoter 1F) and *FKBP5* (intron 7)	Perceived Stress Scale (PSS) and Edinburgh Postnatal Depression Scale (EPDS)	Higher maternal perceived stress was associated with lower *FKBP5* methylation (site 4), which in turn was linked to lower newborn birth weight percentiles.
12	Kocher et al. (2023) [31]	United States (Washington, D.C.)	22 newborns (11 in the pandemic cohort “Project RESCUE”, 11 in the pre-pandemic control cohort)	Full pregnancy (Infants born between April and December 2020)	Maternal exposure to the COVID-19 pandemic (Maternal distress/Prenatal stress). Healthy neonatal development (SARS-CoV-2 negative pregnancies).	Epigenome-wide association study (EWAS) using Illumina MethylationEPIC arrays	Neonatal buccal swabs (collected at birth)	Genome-wide DNA methylation (specifically identifying 58 differentially methylated positions/DMPs)	Maternal stress assessed via the Perceived Stress Scale (PSS) and pandemic exposure status	Newborns exposed to the pandemic in utero showed unique DNA methylation signatures in genes related to neurodevelopment and inflammatory pathways (e.g., *MACROD2*, *PRDM16*)
13	Holdsworth et al. (2023) [32]	United States (New York)	185 mother–child dyads (longitudinal)	Pregnancy (maternal distress assessment) through 7 years postpartum	Maternal prenatal distress (Perceived stress and depressive symptoms). Maternal-infant interaction quality (postnatal environment).	Prospective longitudinal cohort study using bisulfite pyrosequencing and behavioral observation.	Peripheral blood (collected from children at age 7)	DNA methylation of the *NR3C1* gene (specifically the exon 1F promoter, CpG sites 1–13)	Perceived Stress Scale (PSS-10) and Edinburgh Postnatal Depression Scale (EPDS Perceived Stress Scale (PSS) and Center for Epidemiologic Studies Depression Scale (CES-D); Crowell Procedure for interaction quality	Maternal-infant interaction quality at age 7 was significantly associated with *NR3C1* methylation, while prenatal distress was not a significant predictor of methylation at age 7
14	Gultig et al. (2023) [33]	The Netherlands	719 participants (exposed and unexposed to famine)	Prenatal period (specifically divided into Early, Mid, and Late gestation famine exposure)	Prenatal famine exposure (the 1944–45 Dutch Hunger Winter). Posttraumatic Stress Disorder (PTSD) susceptibility in later life.	Retrospective cohort study and Gene x Environment (GxE) interaction analysis	Peripheral blood (for DNA extraction and genotyping)	*NR3C1* (glucocorticoid receptor) gene polymorphisms (BclI, N363S, and GR-9beta)	Watson PTSD Inventory (for PTSD diagnosis and symptoms) and Life Events Checklist (LEC) for trauma exposure	Prenatal famine exposure during mid-gestation, combined with the *NR3C1* BclI G-allele, was associated with higher PTSD symptom severity in later life
15	Santander et al. (2024) [34]	United States (Rhode Island)	143 pregnant people	Second or third trimester (mean 23.11 weeks at assessment; placenta collected at birth)	Maternal prenatal stress (Perceived stress). Socioeconomic status (Household income and Hollingshead Index).	Prospective cohort study using bisulfite conversion, pyrosequencing, and linear regression	Placenta (fetal side)	DNA methylation of *NR3C1* (promoter 1F, CpG sites 1–13) and *FKBP5* (intron 7, CpG sites 1–3)	Perceived Stress Scale (PSS-10)	Prenatal stress was associated with higher *NR3C1* (CpG 8) and *FKBP5* (CpG 1) methylation; lower SES was associated with higher *NR3C1* (CpG 10) and *FKBP5* (CpG 3) methylation
16	Wang et al. (2022) [35]	China (Tangshan)	176 adult subjects (123 prenatally exposed to the earthquake, 53 non-exposed controls)	Prenatal period (categorized by 1st, 2nd, and 3rd trimester exposure during the 1976 Tangshan earthquake)	Prenatal earthquake stress exposure (natural disaster). Long-term working memory (WM) impairment in adulthood.	Retrospective cohort study using Bisulfite Sequencing PCR (BSP) and neurocognitive testing	Peripheral blood (collected from adults aged 42–43)	DNA methylation of the *NR3C1* gene (specifically the exon 1F promoter, 39 CpG sites)	Hopkins Verbal Learning Test-Revised (HVLT-R) and Brief Visuospatial Memory Test-Revised (BVMT-R) for working memory	Prenatal earthquake exposure, especially during the second trimester, was associated with higher *NR3C1* methylation and poorer verbal/visuospatial working memory in adulthood
17	McKenna et al. (2021) [36]	Australia (Queensland)	381 mother–child dyads (longitudinal cohort)	Pregnancy (maternal stress assessed at early, mid, and late pregnancy)	Maternal prenatal perceived stress (MPPS). Offspring depression in early adulthood (age 20).	Prospective longitudinal study using Multilocus Genetic Profile Scores (MGPS) and interaction analysis	Peripheral blood or saliva (offspring DNA at age 20)	HPA-axis polygenic risk: SNPs in *CRHR1* (rs242924), *NR3C1* (rs6198), and *FKBP5* (rs9296158)	Perceived Stress Scale (PSS) for mothers; Diagnostic Interview Schedule for Children (DISC-IV) and Composite International Diagnostic Interview (CIDI) for offspring depression	Higher prenatal stress was associated with more depressive symptoms at age 20 only in offspring with a high HPA-axis polygenic risk score (MGPS)
18	Wang et al. (2018) [37]	Singapore	140 neonates (from the GUSTO cohort)	26 weeks gestation (maternal assessment)	Antenatal maternal depressive symptoms. Neonatal brain morphology (specifically cortical thickness).	Prospective longitudinal study using high-resolution Structural Magnetic Resonance Imaging (sMRI) and genetic interaction analysis	Brain (neonatal imaging) and Umbilical cord blood (for genotyping)	*FKBP5* gene single nucleotide polymorphism (SNP) rs1360780	Perceived Stress Scale (Edinburgh Postnatal Depression Scale (EPDS) for mothers	Prenatal stress predicted higher methylation at CpG8; lower household income was associated with higher methylation at CpG2, 5, and 6 *FKBP5* genotype moderated the effect of maternal depression on neonatal cortical thickness; specifically, carriers of the rs1360780 T allele showed thinner right frontal cortex in response to high maternal depression
19	Mulligan et al. (2012) [38]	Democratic Republic of Congo (DRC)	25 mother–newborn dyads	Throughout pregnancy (mothers assessed at delivery regarding their experience during the preceding pregnancy)	Extreme maternal psychosocial stress (war-related stressors, sexual violence, and social neglect). Newborn birth weight (fetal growth).	Cross-sectional prospective study using bisulfite conversion and pyrosequencing	Umbilical cord blood and Placenta	DNA methylation of the *NR3C1* gene (specifically the exon 1F promoter, 39 CpG sites)	Culturally relevant stress scales (War Trauma, Sexual Violence, and Social Quality scales)	Higher maternal stress (specifically war trauma and social quality) was significantly associated with increased *NR3C1* methylation and lower newborn birth weight
20	Yehuda et al. (2014) [39]	United States (New York)	80 Holocaust offspring and 15 comparison subjects	Preconception (Parental trauma occurred during the Holocaust, prior to the birth of the offspring)	Parental Posttraumatic Stress Disorder (PTSD). Intergenerational transmission of trauma.	Case–control study using bisulfite conversion and pyrosequencing	Peripheral blood (leukocytes) from adult offspring	DNA methylation of the *NR3C1* gene (specifically the exon 1F promoter, CpG sites 1–22)	Clinician-Administered PTSD Scale (CAPS) and SCID; Parental PTSD assessed via the Family History Questionnaire	Maternal and paternal PTSD had opposite effects on offspring *NR3C1* methylation: maternal PTSD was associated with lower methylation, while paternal PTSD was associated with higher methylation
21	Montirosso et al. (2020) [40]	Italy	150 mother–infant dyads (Planned/Estimated)	Post-delivery/Early infancy (focus on infants with neurodevelopmental disabilities born at various gestational ages)	Neurodevelopmental Disabilities (NDDs) in infants (e.g., cerebral palsy, genetic syndromes). Maternal stress and psychological well-being.	Multicentre Randomized Controlled Trial (RCT) protocol	Infant saliva	*DNA* methylation of the *NR3C1* (glucocorticoid receptor) and *SLC6A4* (serotonin transporter) genes	Still-Face Paradigm (SFP) and salivary cortisol reactivity (AUCi)	(Study Protocol) Aims to evaluate if a Video-Feedback Intervention (VIG-plus) reduces infant stress reactivity and modifies DNA methylation compared to standard care
22	Needham et al. (2015) [41]	United States	1264 participants (Multi-Ethnic Study of Atherosclerosis—MESA)	Life course (includes childhood SES, representing the early postnatal/developmental period, and adult SES)	Socioeconomic status (SES) across the life course (Childhood and Adult). Risk for cardiovascular disease and chronic inflammation.	Multi-level modeling of DNA methylation data from the Illumina HumanMethylation450 BeadChip	Peripheral blood (purified monocytes)	DNA methylation of 18 genes related to stress reactivity (*NR3C1*, *AVP*, *CRH*, *FKBP5*, etc.) and inflammation (*IL6*, *TNF*, *NFKB1*, etc.)	Life course SES measures (Childhood SES based on parental education; Adult SES based on own education and neighborhood wealth)	Low childhood SES was associated with lower DNA methylation in stress reactivity genes and higher methylation in inflammation genes, suggesting biological embedding of early-life social disadvantage
23	Lin et al. (2022) [42]	China	513 pairs of parents and infants	Third trimester (maternal assessment) and placenta collected at delivery	Maternal depression, anxiety, and sleep quality during pregnancy. Infant sleep disorder (within 3 months after birth)	Prospective cohort study using immunohistochemistry and next-generation sequencing-based bisulfite sequencing PCR (nBS-PCR)	Placenta	Protein expression and DNA methylation of *NR3C1*, *NR3C2* (Glucocorticoid), *MTNR1A*, *MTNR1B* (Melatonin), *RASGRF1*, *RASGRF2* (EPAC), *DRD1*, and *DRD2* (Dopamine)	Self-rating Depression Scale (SDS), Self-rating Anxiety Scale (SAS), and Pittsburgh Sleep Quality Index (PSQI) for mothers	Maternal prenatal emotion and sleep quality were associated with infant sleep; specifically, *NR3C1* and *MTNR1A* methylation/expression in the placenta mediated these effects
24	Duis et al. (2018) [43]	United States (Boston)	436 mother–infant dyads	Pregnancy (maternal history and clinical diagnosis assessed at recruitment)	Maternal affective disorder (Major Depressive Disorder—MDD and/or Anxiety). Preterm birth and low birth weight (covariates in the analysis).	Prospective cohort study using the Sequenom MassARRAY EpiTYPER system	Umbilical cord blood (neonatal)	DNA methylation of the *FKBP5* gene (specifically Intron 7, involving 6 CpG sites)	Maternal clinical diagnosis of MDD/Anxiety (as a proxy for prenatal stress) and *FKBP5* genotype	Maternal affective disorder was associated with lower *FKBP5* methylation in neonates; this effect was significantly moderated by the infant’s *FKBP5* genotype (rs1360780)
25	Bleker et al. (2020) [44]	Australia	23 children (offspring of mothers originally randomized into 12 CBT and 11 TAU)	Antenatal (pregnancy) during the intervention; children assessed 3–7 years after birth	Antenatal maternal depression. Long-term neurobiological, behavioral, and cognitive development of offspring.	Perspective article summarizing a pilot Randomized Controlled Trial (RCT) and its follow-up	Buccal swabs (for DNA methylation) and Saliva (for cortisol)	Genome-wide DNA methylation; *NR3C1* (glucocorticoid receptor) gene; Salivary cortisol	Salivary cortisol response to a standardized stressor (Modified Trier Social Stress Test for Children—TSST-C)	No significant differences were found between treatment groups (CBT vs. TAU) in DNA methylation, cortisol reactivity, or cognitive/behavioral scores
26	Chen et al. (2014) [45]	United States (New York)	100 mother–infant dyads	Throughout pregnancy (maternal stress assessed via questionnaires regarding the pregnancy period)	Maternal prenatal stress (Self-reported perceived stress, anxiety, and stressful life events). Clinical diagnosis of Depression or Anxiety disorder in the mother.	Comparative epigenetic study using bisulfite conversion and pyrosequencing	Placenta and Umbilical cord blood (Comparison of both tissues)	DNA methylation of stress-related genes: *NR3C1*, *NR3C2*, *SLC6A4*, *HSD11B2*, *CRHR1*, *CRHR2*, and *IGF2/H19*	Perceived Stress Scale (PSS), State-Trait Anxiety Inventory (STAI), and Stressful Life Events (SLE)	Maternal stress and clinical mood disorders were associated with distinct DNA methylation patterns, with the placenta showing more significant associations than cord blood
27	King et al. (2016) [46]	United States (North Carolina)	296 mother–infant dyads (from the NEST cohort)	Pregnancy (maternal environment assessed during pregnancy; cord blood collected at birth)	Neighborhood disadvantage (socioeconomic environment). Family environment/social support (e.g., presence of the mother’s mother).	Prospective cohort study using Hierarchical Linear Modeling (HLM) and bisulfite pyrosequencing	Umbilical cord blood	DNA methylation of the *MEG3* (Maternally Expressed 3) Differentially Methylated Region (DMR)	Neighborhood Disadvantage Index (census-based); maternal relationship status; and social support indicators	Higher neighborhood disadvantage and lack of social support (e.g., absence of the maternal grandmother) were associated with increased *MEG3* methylation in newborns
28	Hogg et al. (2013) [47]	Canada	167 placental samples (including 111 controls, 19 early-onset pre-eclampsia, 18 late-onset pre-eclampsia, and 19 normotensive intrauterine growth restriction)	Late second to third trimester (samples collected at delivery; early-onset cases 34 weeks)	Early-onset pre-eclampsia (EOPET). Late-onset pre-eclampsia (LOPET) and Intrauterine Growth Restriction (IUGR)	Case–control study using Illumina HumanMethylation450 BeadChip array and bisulfite pyrosequencing	Placenta (chorionic villi)	DNA methylation of *HSD11B2*, *NR3C1*, *FKBP5*, *SERPINA6*, *CYP11A1*, and *CYP19A1*	Clinical diagnosis of pre-eclampsia (maternal hypertensive disorder)	Early-onset pre-eclampsia is associated with significantly lower *HSD11B2* and *CYP19A1* methylation, and higher *NR3C1* and *FKBP5* methylation in the placenta
29	Cao-Lei et al. (2015) [48]	Canada (Québec)	34 adolescents (whose mothers were pregnant during the 1998 Ice Storm)	Prenatal (various trimesters during the January 1998 Ice Storm)	Prenatal Maternal Stress (PNMS) following a natural disaster. Long-term immune function and health outcomes in adolescence	Prospective longitudinal study; genome-wide DNA methylation using Illumina HumanMethylation450 BeadChip	Peripheral blood (specifically T cells) collected at age 13	Genome-wide DNA methylation (2872 specific CpG sites identified)	Questionnaire of Objective Stress (Storm 32); Impact of Event Scale-Revised (IES-R); Cognitive Appraisal (positive vs. negative)	Maternal negative cognitive appraisal of the disaster was associated with distinct DNA methylation patterns in 1564 genes, primarily involved in immune system pathways
30	Dereix et al. (2021) [49]	United States (Boston, Massachusetts)	200 pregnant women	Second trimester (assessment at 24–28 weeks gestation)	Maternal prenatal anxiety. Maternal prenatal depression	Prospective cohort study using bisulfite conversion and pyrosequencing	Umbilical cord blood (collected at delivery)	DNA methylation of the *NR3C1* gene (specifically the exon 1F promoter, 13 CpG sites)	Edinburgh Postnatal Depression Scale (EPDS) and State-Trait Anxiety Inventory (STAI)	Higher maternal anxiety was significantly associated with lower *NR3C1* methylation (CpG site 10); no significant association was found for maternal depression
31	Cardenas et al. (2019) [50]	United States (Project Viva, Massachusetts) and the Netherlands (Generation R, Rotterdam)	Discovery: 465 (Project Viva); Replication: 978 (Generation R); Total: 1443 mother–infant dyads	Pregnancy (Antidepressant use and mood symptoms assessed during the 1st and 2nd trimesters)	Maternal prenatal antidepressant use (SSRIs). Maternal prenatal anxiety and depression.	Epigenome-wide association study (EWAS) and longitudinal persistence analysis	Umbilical cord blood (at birth) and peripheral blood (childhood follow-up at ages 3.3 and 7.7 years).	Genome-wide DNA methylation (Infinium HumanMethylation450 BeadChip)	Edinburgh Postnatal Depression Scale (EPDS) and State-Trait Anxiety Inventory (STAI)	Prenatal antidepressant use was associated with DNA methylation changes at birth (e.g., *CHRNBP2*, *CYP1A1*), but these associations did not persist into early or mid-childhood
32	Stroud et al. (2024) [51]	United States (Rhode Island)	198 pregnant women	Pregnancy (mean gestational age at assessment: 25 weeks; placenta collected at birth)	Lifetime trauma exposure (Preconception). Perinatal PTSD (Post-Traumatic Stress Disorder) symptoms	Pilot study using bisulfite conversion and pyrosequencing to analyze DNA methylation	Placenta	DNA methylation of *NR3C1* (promoter 1F) and *FKBP5* (intron 7)	Life Events Checklist (LEC) for trauma; Posttraumatic Diagnostic Scale (PDS-5) for PTSD symptoms	PTSD symptoms were associated with higher *NR3C1* methylation (site 10) and lower *FKBP5* methylation (site 2); lifetime trauma without symptoms showed lower *NR3C1* methylation (site 5)
33	Serpeloni et al. (2017) [52]	Brazil (Rio de Janeiro)	121 children (the third generation/grandchildren)	Prenatal period of the mother (when the grandmother was pregnant with the child’s mother)	Grandmaternal exposure to interpersonal violence (IPV) during pregnancy. Multigenerational transmission of trauma and health outcomes in grandchildren.	Epigenome-wide association study (EWAS) using Illumina HumanMethylation450 BeadChip	Peripheral blood (from the grandchildren)	Genome-wide DNA methylation (identifying 5 specific CpG sites: *GABBR1*, *NEK1*, *WDR82*, *C19orf21*, and *CHST11*)	Conflict Tactics Scale (Revised) to assess grandmaternal interpersonal violence (physical and sexual)	Grandmaternal stress during pregnancy was associated with unique DNA methylation signatures in the grandchildren, suggesting a multigenerational impact
34	Bleker et al. (2019) [53]	Australia	23 children (12 in the CBT group, 11 in the TAU group)	Pregnancy (mothers received treatment during pregnancy; children assessed at 3–7 years of age)	Antenatal maternal depression. Long-term neurodevelopmental outcomes in offspring.	Follow-up of a pilot Randomized Controlled Trial (RCT) using the Illumina MethylationEPIC BeadChip	Buccal swabs (childhood)	Genome-wide DNA methylation (770,668 CpG sites) and 16 candidate genes (e.g., *NR3C1*, *FKBP5*, *SLC6A4*, *BDNF*)	Maternal depression assessed via the Beck Depression Inventory (BDI-II) and Beck Anxiety Inventory (BAI)	No significant differences in DNA methylation were found between children whose mothers received Cognitive Behavioral Therapy (CBT) versus Treatment as Usual (TAU)

#### 3.2.4. Compensatory Buffering and the Reversibility of Epigenetic Damage

A crucial aspect emerging from this synthesis is the identification of compensatory mechanisms, such as the hypermethylation of the serotonin transporter gene (SLC6A4), which may function as a biological buffer to attenuate the impact of elevated maternal cortisol on neonatal reactivity. This dimorphism suggests that the impact of prenatal stress follows sex-specific developmental trajectories that determine long-term behavioral vulnerability. Findings regarding Cognitive Behavioral Therapy (CBT) and postnatal care interventions show that the normalization of NR3C1 methylation levels is possible, providing biological evidence for the efficacy of psychotherapeutic support. Identifying specific placental methylation profiles or markers of “epigenetic aging” (such as telomere shortening) could therefore serve as a transformative early screening tool to detect infants at risk for preterm birth or neurodevelopmental disorders, facilitating targeted preventive medicine.

### 3.3. Quality Assessment Results

Regarding the methodological quality assessment conducted using the Newcastle–Ottawa Scale (NOS), the systematic analysis reveals an extremely high level of scientific rigor among the studies included in this work. As detailed in Table 4, all 34 examined records were classified as “High Quality,” reflecting a robust structural framework in the phases of selection, comparability, and outcome ascertainment.

Specifically, nearly all studies obtained the maximum score in the Selection (4 stars) and Comparability (2 stars) categories, demonstrating effective control of confounding variables and adequate representativeness of the analyzed samples. Concerning the Outcome assessment, the majority of the works reached the top of the scale with 3 stars, with only a few rare exceptions that nevertheless maintained an optimal qualitative standard. Consequently, the Risk of Bias was found to be uniformly low (“L”) across the entire bibliographic corpus, ensuring the reliability of the molecular and clinical evidence synthesized in this review.

### 3.4. Results of Syntheses

#### 3.4.1. Overall Effect Estimate and Moderator Analysis

**Figure 2:**
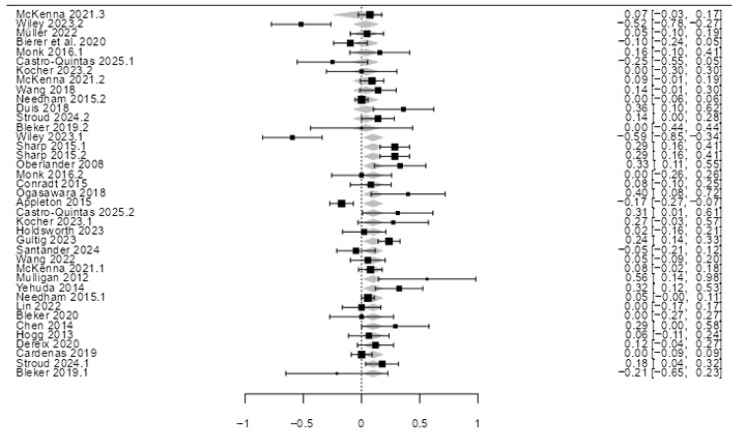
Forest plot of the meta-analysis showing the association between genetic determinants and stress reactivity in pregnancy. The square points represent the log Risk Ratio (log RR) for each study, and the diamond represents the pooled estimate [20,21,22,23,24,25,26,27,28,30,31,32,33,34,35,36,37,38,39,41,42,43,44,45,47,49,50,51,53].

The meta-analysis was conducted on a sample of k = 39 studies using a mixed-effects model. Between-study variance was estimated using the restricted maximum-likelihood (REML) estimator, with the Knapp and Hartung (2003) adjustment applied. The model intercept yielded an estimate of 0.1623 (SE = 0.0897; 95% CI [−0.019, 0.344]). While a trend toward a positive correlation between genetic determinants and stress reactivity was observed, the effect did not reach formal statistical significance (Z = 1.81, *p* = 0.079), as visualized in the Forest Plot (Figure 2). The inclusion of a moderator variable did not produce a statistically significant coefficient (beta = −0.0628; SE = 0.0610; Z = −1.03, *p* = 0.310), indicating that this specific predictor does not significantly account for the observed between-study variability.

#### 3.4.2. Heterogeneity Analysis and Model Fit

The analysis revealed a substantial level of statistical heterogeneity across the included comparisons (Q(38) = 175.76, *p* < 0.0001; I^2^ = 84.07%; tau^2^ = 0.2319). This high degree of variance is likely attributable to the significant methodological diversity inherent in the primary studies, particularly concerning the variety of biological tissues sampled—ranging from placental tissue and umbilical cord blood to maternal and infant saliva—and the diverse psychometric instruments employed to quantify maternal stress phenotypes. While *NR3C1*, *FKBP5*, and *CRHR1* were all identified as key genetic candidates during the systematic search, the quantitative meta-analysis focused predominantly on *NR3C1* and *FKBP5*. Quantitative data for *CRHR1* were found to be insufficient for a robust meta-analytic synthesis due to the limited number of studies reporting compatible effect sizes. Consequently, the role of *CRHR1* was addressed through qualitative systematic synthesis, whereas the mixed-effects model was utilized to statistically account for the observed variance among the more frequently reported markers.

#### 3.4.3. Publication Bias Assessment and *p*-Curve Analysis

**Figure 3:**
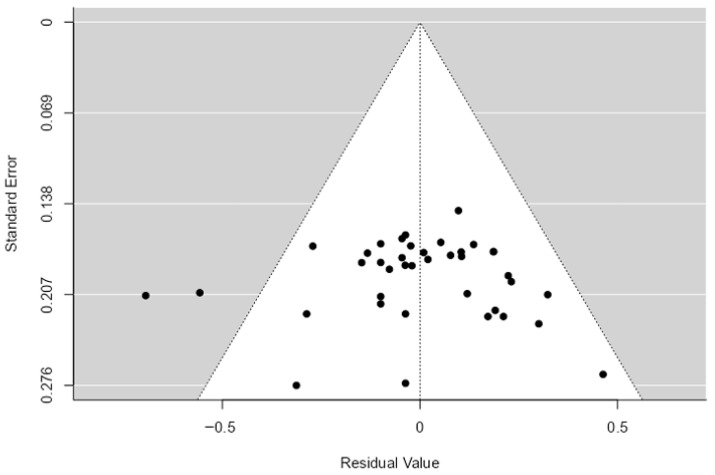
Funnel plot of standard error by residual value. The symmetrical distribution of study results around the vertical mean indicates high resilience to publication bias.

To exclude systematic distortions and evaluate the stability of the pooled estimates, several sensitivity tests were performed. Visual inspection of the Funnel Plot (Figure 3) showed a symmetrical distribution of residuals, suggesting the absence of significant publication bias. This observation was statistically confirmed by asymmetry tests, including the Begg and Mazumdar rank correlation (*p* = 0.809) and Egger’s regression (*p* = 0.915). Furthermore, Rosenthal’s Fail-Safe N was 431,000 (*p* < 0.001), indicating that the findings are robust against potential unpublished studies. To further support the evidentiary value, *p*-curve and *p*-uniform analyses were utilized to estimate the corrected effect size and evaluate potential significance inflation. The results indicate that the distribution of significant *p*-values is not biased by selection practices, thereby confirming the scientific integrity of the dataset.

#### 3.4.4. Outlier and Influence Diagnostics

The reliability of the estimates was consolidated through a rigorous analysis of influential cases. Examination of externally standardized residuals, Cook’s distance, and DFFITS values identified no studies capable of disproportionately altering the model intercept. The leave-one-out procedure for tau estimates and the Q test confirmed the stability of the heterogeneity structure. Finally, the Q-Q plot demonstrated a residual distribution consistent with the normality assumptions of the mixed-effects model.

## 4. Discussion

### 4.1. Genomic Architecture, Statistical Power, and the Biological Embedding of Stress

The present systematic review aggregates data from 39 independent studies, providing a critical mass of evidence that overcomes the statistical power limitations inherent in single neonatal cohorts, which are often numerically small and subject to confounding variables that limit generalizability [53,54,55]. The robustness of the meta-analytic estimates presented herein, supported by a Rosenthal Fail-Safe N of 431,000 (*p* < 0.001), confirms that the relationship between genetic determinants and stress reactivity is not a stochastic statistical fluctuation, but a biological constant resilient to publication bias [56].

A pivotal finding of this analysis is the identification of a critical vulnerability window during the third gestational trimester. In this phase, maternal stress does not act as a transient insult but initiates a process of biological embedding. This process is fueled by the fact that the fetus is capable of synthesizing cortisol *de novo* by mid-pregnancy and possesses a fully formed adrenal zona fasciculata by the third trimester, providing the basis for a fetal stress response independent of the mother [14].

The intrauterine biochemical environment, saturated by maternal pro-inflammatory signals and glucocorticoids, is transduced into molecular signals that “write” the fetus’s biology through stable epigenetic modifications. This imprinting is not limited to recalibrating the stress response “thermostat” but induces an acceleration of the neonatal epigenetic clock at birth. Furthermore, environmental stressors can trigger the shortening of neonatal telomeres, which serve as early biomarkers of biological aging and may reduce overall fetal longevity [7]. Such precocious aging provides a mechanistic explanation for the “fetal programming” theory, justifying the early onset of chronic metabolic and cardiovascular pathologies in adulthood among individuals exposed to prenatal stress [1,38,57].

While previous evidence underscores that the psychophysical imbalance typical of post-traumatic stress disorder (PTSD) is significantly associated with an increased risk of cardiovascular diseases, our synthesis reinforces the link between these molecular scars and long-term systemic vulnerability [58]. This systemic vulnerability is further exacerbated in preterm infants, who are nearly 18 times more likely to experience repeated hospitalizations during childhood compared to those born at term [14].

Our results establish a statistically robust foundation confirming that fetal programming is a non-random process mediated by late-gestational stress. Furthermore, this analysis demonstrates that the acceleration of the neonatal epigenetic clock serves as a primary mediator between an adverse intrauterine environment and long-term systemic health outcomes.

### 4.2. Mechanistic Mapping: Gene Cross-Talk and the Molecular “Double Hit”

At the core of this discussion lies the understanding of the cross-talk (molecular dialog) between the genomic loci governing the HPA axis.

This analysis reveals that neonatal allostatic load is not determined by a single gene but by a synergistic interaction that defines the organism’s adaptive capacity. The glucocorticoid receptor (*NR3C1*) and its chaperone regulator (*FKBP5*) form an essential ultradian feedback loop for homeostasis; however, hypermethylation of the *NR3C1* promoter reduces receptor density while the *FKBP5* rs1360780 risk variant further depresses binding affinity. This interaction is critical because FKBP5 gene variants maintain the receptor in a high-affinity state; variations in this specific mechanism can impede the effective dampening of cortisol release following stress [5]. Clinically, this molecular “double hit” generates chronic glucocorticoid resistance, causing the HPA axis to lose its ability to “shut off” via negative feedback and exposing the developing fetal brain to neurotoxic levels of cortisol.

This process provides a mechanical explanation for hippocampal volume reduction and intrauterine growth restriction (IUGR), as excess cortisol inhibits cellular proliferation and placental glucose metabolism [35,43,45,51]. For accurate clinical interpretation, it is essential to distinguish between acute stress reactivity and basal fluctuations, as the bioactive fraction of salivary cortisol serves as a reliable mirror of the free response in the bloodstream [5]. A compelling finding further concerns sex-specific susceptibility, suggesting that male and female placentae respond to prenatal stress through divergent trajectories. Males tend to exhibit greater immediate epigenetic sensitivity, utilizing growth reduction as a survival strategy—a “vulnerable” profile—whereas females show greater growth stability but more pronounced epigenetic alterations in anxiety-related circuits, which manifest as long-term behavioral vulnerability. Consequently, fetal sex acts as a critical biological moderator that must be integrated into precision medicine protocols [45,59].

Based on these findings, we identify the synergistic interaction between NR3C1 and FKBP5 as the central molecular mechanism driving neonatal glucocorticoid resistance. Additionally, our study establishes that fetal sex acts as a critical biological moderator that determines whether stress vulnerability manifests as physical growth deficits or long-term behavioral predisposition.

### 4.3. The Placental–Brain Axis and Differential Susceptibility

The placenta emerges not merely as a filter but as a pacemaker for neurological development. Due to a shared ectodermal origin, the placenta serves as an “archive” of the intrauterine stress environment; as a non-article related example, placental *MTNR1A* methylation “predicts” circadian rhythm dysregulation and neonatal sleep disturbances in the first months of life [25,42,60]. In this context, maternal resilience functions as a biological pacemaker, optimizing the negative feedback system to balance receptors and prevent exacerbated responses such as fear or depression [7].

However, the most significant theoretical advancement is the shift from the “Diathesis-Stress” model toward Differential Susceptibility. The epigenetic alterations described here, such as *SLC6A4* hypermethylation, are not merely “scars” or damages but indicators of increased environmental permeability. Neonates with these signatures act as “orchid children”: extremely fragile in adverse contexts but possessing superior repair plasticity when placed in supportive environments. An individual’s capacity for flexibility and competence can mitigate the impact of stressors, suggesting that social support acts as a powerful protective buffer in this dynamic [7]. Targeted bio-behavioral interventions, such as video-feedback (*VIG-plus*), leverage this window of plasticity to induce positive epigenetic “reprogramming,” stabilizing the child’s health trajectory up to seven years post-partum and transforming a risk condition into an opportunity for clinical excellence and resilience [29,40].

In summary, our data support the redefinition of epigenetic modifications as markers of biological plasticity rather than simple indicators of damage. This shift suggests that early therapeutic interventions have the biological potential to reverse risk trajectories, effectively transforming vulnerability into an opportunity for resilience.

### 4.4. Methodological Challenges and the Future of Precision Prenatal Medicine

Despite the consistency of the data, research in behavioral epigenetics faces significant methodological challenges. The heterogeneity of sampled tissues (saliva, cord blood, peripheral blood) and the temporal stability of samples require rigorous standardization of collection and analysis protocols [29]. To correctly disentangle reactivity from recovery, physiologically plausible models are required that account for the secretory delays inherent in the synthesis of cortisol from cholesterol [5]. Furthermore, the integration of Polygenic Risk Scores (PRS) with tissue-specific biomarkers represents the necessary frontier to overcome current diagnostic fragmentation. Understanding the dialog between genes and the environment provides the tools to interrupt the chain of intergenerational trauma transmission, safeguarding not only the neonate but the health of future generations through personalized, stratified, and timely preventive interventions [19,20,21,22,23,24,25,26,27,28,29,30,31,32,33,34,35,36,37,38,39,40,41,42,43,44,45,46,47,48,49,50].

Our findings indicate that the future of precision prenatal medicine lies in the integration of epigenetic profiles with polygenic risk scores. This integrated approach is essential for transitioning from reactive care to proactive prevention strategies that protect the integrity of the mother–infant dyad.

### 4.5. Study Limitations

Despite the clinical relevance of these findings, several methodological limitations must be acknowledged. First, the observed statistical heterogeneity (I^2^ = 84.07%) represents a significant challenge for the standardization of results. This variability reflects the biological complexity of prenatal programming, as the included studies utilized different biological matrices and evaluated distinct stress dimensions (e.g., general anxiety vs. pregnancy-specific stress). While epigenetic changes in offspring remain a crucial factor, they are still understudied in the specific context of preterm birth associated with maternal stress [14]. Furthermore, the frequent use of cross-sectional designs precludes the establishment of definitive causality and does not allow for the complete exclusion of confounding variables, such as nutritional factors or environmental toxicant exposures.

A further limitation concerns the availability of data for specific genetic markers; for instance, the scarcity of compatible quantitative metrics for the *CRHR1* gene limited its inclusion in the meta-analytic model, necessitating a more descriptive approach for this specific locus. Additionally, there is a notable lack of sex-disaggregated data in many primary studies, which is critical given the known sex-dependent susceptibility of the fetus to intrauterine insults. Finally, while the current evidence provides strong short-term insights, there remains a shortage of long-term longitudinal studies capable of tracking the persistence of these epigenetic signatures from birth into late adulthood.

The limitations highlighted here emphasize the urgent need for methodological standardization to reduce outcome heterogeneity in future research. Our work underscores that future studies must prioritize sex-disaggregated analysis and extended longitudinal monitoring to validate the temporal stability of the identified biomarkers.

## 5. Conclusions

This systematic review and meta-analysis demonstrate that maternal genetic predisposition, particularly within the HPA axis regulatory genes, plays a pivotal role in modulating stress reactivity during pregnancy. Our findings shift the focus from a purely environmental perspective to a gene-environment interaction framework, suggesting that certain genetic variants can significantly amplify the biological impact of prenatal distress on both the mother and the developing fetus.

From a clinical standpoint, these results underscore the necessity of integrating genetic markers into prenatal screening protocols. Identifying women with high genetic vulnerability to stress could allow for personalized early interventions, potentially mitigating the long-term risks associated with fetal programming, such as neurodevelopmental disorders and altered metabolic trajectories.

In conclusion, while this study confirms a significant association between specific genotypes and stress-related biomarkers, further longitudinal research is required to fully map the epigenetic mechanisms involved. Ultimately, bridging the gap between genomic medicine and obstetric care is essential for developing targeted strategies that protect the biological and psychological health of the mother–infant dyad.

## Figures and Tables

**Table 1:** Summary of eligibility criteria based on the PICOS framework.

PARAMETER	INCLUSION CRITERIA
**POPULATION**	Pregnant women at any stage of gestation and their offspring (from neonates to early childhood).
**INTERVENTION/EXPOSURE**	Documented maternal stress, including trauma, socioeconomic adversity, stressful life events (SLEs), or clinical psychological distress.
**COMPARISON**	Assessments between genotypic variants (e.g., risk allele carriers vs. wild-type homozygotes) or between cohorts exposed to different levels of environmental stress.
**OUTCOME**	Genetic and epigenetic determinants of the HPA axis (specifically DNA methylation and polymorphisms of *NR3C1*, *FKBP5*, and *CRHR1* genes) in maternal or fetal tissues (placenta, cord blood).
**STUDY DESIGN**	Primary observational studies, including prospective and retrospective cohorts, case–control studies, and cross-sectional analyses.

**Table 2:** Search strategy summary.

Database	Search Terms	Limits/Filters	Results Retrieved	Date of Search
PubMed	(“Pregnancy” OR “Pregnant Women” OR “Prenatal Exposure Delayed Effects” OR pregnancy OR prenatal OR maternal OR antenatal AND Stress, Psychological” OR “Hydrocortisone” OR “Hypothalamo-Hypophyseal System” OR “Pituitary-Adrenal System” OR “Stress reactivity” OR “Cortisol” OR “HPA axis” OR “Stress response”) AND (“Tacrolimus Binding Proteins” OR “FKBP5” OR “Receptors, Corticotropin-Releasing Hormone” OR “CRHR1” OR “CRH-R1” OR “Receptors, Glucocorticoid” OR “NR3C1” OR “Glucocorticoid Receptor”) AND (“Genetic Variation” OR “Polymorphism, Single Nucleotide”OR “Epigenomics” OR “DNA Methylation” OR “Genetic” OR “Polymorphism” OR “SNP” OR “Methylation” OR “Genotype”)	English language only	205	January 2026
Scopus	(“Pregnan” OR “Maternal” OR “Prenatal” OR “Antenatal” OR “Gestation”) AND (“Stress” OR “Reactivity” OR “Cortisol” OR “HPA axis” OR “Hydrocortisone” OR “Stress response”) AND (“FKBP5” OR “CRHR1” OR “NR3C1” OR “Glucocorticoid receptor” OR “Corticotropin-releasing hormone receptor 1”) AND (“Genet” OR “Polymorphism” OR “SNP” OR “Methylation” OR “Epigenet” OR “Genotype”)	English language only	440	January 2026
Web of Science	(“Pregnan” OR “Maternal” OR “Prenatal” OR “Antenatal” OR “Gestation”) AND (“Stress” OR “Reactivity” OR “Cortisol” OR “HPA axis” OR “Hydrocortisone” OR “Stress response”) AND (“FKBP5” OR “CRHR1” OR “NR3C1” OR “Glucocorticoid receptor” OR “Corticotropin-releasing hormone receptor 1”) AND (“Genet” OR “Polymorphism” OR “SNP” OR “Methylation” OR “Epigenet” OR “Genotype”)	English	785	January 2026

**Table 4:** Risk-of-bias evaluation.

Study n.	Study	Selection (Max 4 Stars)	Comparability (Max 2 Stars)	Outcome (Max 3 Stars)	Risk-of-Bias Assessment	Risk of Bias
1	Wiley et al. (2023) [20]	★★★★	★	★★	High Quality	**L**
2	Müller et al. (2022) [21]	★★★★	★★	★★	High Quality	**L**
3	Sharp et al. (2015) [22]	★★★★	★★	★★★	High Quality	**L**
4	Oberlander et al. (2008) [23]	★★★★	★★	★★★	High Quality	**L**
5	Bierer et al. (2020) [24]	★★★★	★★	★★★	High Quality	**L**
6	Monk et al. (2016) [25]	★★★★	★★	★★★	High Quality	**L**
7	Conradt et al. (2015) [26]	★★★★	★★	★★★	High Quality	**L**
8	Ogasawara et al. (2018) [27]	★★★★	★★	★★★	High Quality	**L**
9	Appleton et al. (2015) [28]	★★★★	★★	★★★	High Quality	**L**
10	Provenzi et al. (2020) [29]	★★★★	★★	★★★	High Quality	**L**
11	Castro-Quintas et al. (2025) [30]	★★★★	★★	★★★	High Quality	**L**
12	Kocher et al. (2023) [31]	★★★★	★★	★★★	High Quality	**L**
13	Holdsworth et al. (2023) [32]	★★★★	★★	★★★	High Quality	**L**
14	Gultig et al. (2023) [33]	★★★★	★★	★★★	High Quality	**L**
15	Santander et al. (2024) [34]	★★★★	★★	★★★	High Quality	**L**
16	Wang et al. (2022) [35]	★★★★	★★	★★★	High Quality	**L**
17	McKenna et al. (2021) [36]	★★★★	★★	★★★	High Quality	**L**
18	Wang et al. (2018) [37]	★★★★	★★	★★★	High Quality	**L**
19	Mulligan et al. (2012) [38]	★★★★	★★	★★★	High Quality	**L**
20	Yehuda et al. (2014) [39]	★★★★	★★	★★★	High Quality	**L**
21	Montirosso et al. (2020) [40]	★★★★	★★	★★★	High Quality	**L**
22	Needham et al. (2015) [41]	★★★★	★★	★★★	High Quality	**L**
23	Lin et al. (2022) [42]	★★★★	★★	★★★	High Quality	**L**
24	Duis et al. (2018) [43]	★★★★	★★	★★★	High Quality	**L**
25	Bleker et al. (2020) [44]	★★★★	★★	★★★	High Quality	**L**
26	Chen et al. (2014) [45]	★★★★	★★	★★★	High Quality	**L**
27	King et al. (2016) [46]	★★★★	★★	★★★	High Quality	**L**
28	Hogg et al. (2013) [47]	★★★★	★★	★★★	High Quality	**L**
29	Cao-Lei et al. (2015) [48]	★★★★	★★	★★★	High Quality	**L**
30	Dereix et al. (2021) [49]	★★★★	★★	★★★	High Quality	**L**
31	Cardenas et al. (2019) [50]	★★★★	★★	★★★	High Quality	**L**
32	Stroud et al. (2024) [51]	★★★★	★★	★★★	High Quality	**L**
33	Serpeloni et al. (2017) [52]	★★★★	★★	★★	High Quality	**L**
34	Bleker et al. (2019) [53]	★★★★	★★	★★	High Quality	**L**

## Data Availability

The data presented in this study are available on request from the corresponding authors due to privacy.
